# A comprehensive pan-cancer analysis unveiling the oncogenic effect of plant homeodomain finger protein 14 (PHF14) in human tumors

**DOI:** 10.3389/fgene.2023.1073138

**Published:** 2023-03-10

**Authors:** Zhiyou Cao, Haibo Zhan, Weiwei Wu, Zhihui Kuang, Fengbo Mo, Xuqiang Liu, Min Dai

**Affiliations:** ^1^ Department of Orthopedics, The First Affiliated Hospital of Nanchang University, Nanchang, Jiangxi, China; ^2^ Artificial Joints Engineering and Technology Research Center of Jiangxi Province, Nanchang, Jiangxi, China

**Keywords:** PHF14, pan-cancer analysis, prognosis, carcinogenesis, cancer, gene expression

## Abstract

The plant homeodomain (PHD) finger refers to a protein motif that plays a key role in the recognition and translation of histone modification marks by promoting gene transcriptional activation and silencing. As an important member of the PHD family, the plant homeodomain finger protein 14 (PHF14) affects the biological behavior of cells as a regulatory factor. Several emerging studies have demonstrated that PHF14 expression is closely associated with the development of some cancers, but there is still no feasible pan-cancer analysis. Based on existing datasets from the Cancer Genome Atlas (TCGA) and the Gene Expression Omnibus (GEO), we performed a systematic analysis of the oncogenic role of the PHF14 gene in 33 human cancers. The expression level of PHF14 was significantly different between different types of tumors and adjacent normal tissues, and the expression or genetic alteration of PHF14 gene was closely related to the prognosis of most cancer patients. Levels of cancer-associated fibroblasts (CAFs) infiltration in various cancer types were also observed to correlate with PHF14 expression. In some tumors, PFH14 may play a role in tumor immunity by regulating the expression levels of immune checkpoint genes. In addition, the results of enrichment analysis showed that the main biological activities of PHF14 were related to various signaling pathways or chromatin complex effects. In conclusion, our pan-cancer research shows that the expression level of PHF14 is closely related to the carcinogenesis and prognosis of certain tumors, which needs to be further verified by more experiments and more in-depth mechanism exploration.

## Introduction

Histones, as an essential protein in eukaryotic chromatin and prokaryotic cells, couples with DNA to form nucleosome structure. Histone recognition figures prominently in cell division and development, gene expression and chromatin organization. Plant homeodomain (PHD), a protein motif in eukaryotes from yeast to humans, exerts epigenetic regulation by reading histone states and functions in various biological events (Aasland et al., 1995; Lu et al., 2018). However, PHD promotes gene transcriptional activation and silencing through differential recognition of methylated or unmodified lysine, leading to histone modification mark recognition and translation (Wysocka et al., 2006; Lan et al., 2007). Plant homeodomain finger protein 14 (PHF14), a multi-PHD finger protein found recently, is an important member of the PHD family. PHF14 is encoded by a highly conserved gene found on chromosome 19p13.2 and consists of approximately 500–900 residues, including 3 to 4 PHD finger modules, which primarily interacts with histones through its PHD1 and PHD3 domains (Huang et al., 2013; Zheng et al., 2021).

Notably, most of the family members referred to as PHD have been previously identified with high involvement a wide range of diseases, including malignancies. For example, the dysregulation of PHF1, PHF3, PHF5, PHF10, PHF11 and PHF20 has been repeatedly reported to lead to neurological diseases, immunodeficiency or cancer (Reader et al., 2007; Hubert et al., 2013; Hiddingh et al., 2014; Mudbhary et al., 2014; Long et al., 2018). In addition, loss of PHF6 has been shown to enhance the activity of tumor-initiating leukemia stem cells in lymphocytic leukemia (Wendorff et al., 2019). PHF8 can promote the progression of gastric and prostate cancer (Li et al., 2017; Liu et al., 2021). PHF19 drives the proliferation of hepatocellular carcinoma and glioblastoma cell (Deng et al., 2018; Xiaoyun et al., 2021). Despite the close association between some members of the PHD finger family and multiple tumors, PHF14 has been poorly studied, resulting in its role in numerous tumors to be explored.

Nevertheless, we can still find from several previous studies that PHF14, as a newly discovered regulator, can be used not only as a transcriptional regulator of target gene expression (Soliman and Riabowol, 2007; Sanchez and Zhou, 2011), but also as a novel epigenetic regulator of hypoxia sensitivity of cell proliferation (Huang et al., 2013; Pan et al., 2022). Therefore, some scholars believe that PHF14 may be related to tumorigenesis and development. For example, it is believed that, PHF14, as an inhibitor, can not only improve colon cancer (Huang et al., 2013; Pan et al., 2022), but also play an important role in biliary tract cancer (BTC). Namely, its over-expression can effectively inhibit the growth of tumor cells (Huang et al., 2013; Pan et al., 2022). Haploinsufficiency or overexpression of PHF14 may interfere with the regulation of neuronal differentiation and development. On this basis, PHF14 is also associated with Dandy-Walker syndrome (Liao et al., 2012). In addition, PHF14 depletion has been revealed to inhibit lung and bladder cancer cell proliferation and tumorigenesis (Huang et al., 2013; Pan et al., 2022). It was also found that the loss of PHF14 would lead to the inhibition of the respiratory system (Huang et al., 2013; Pan et al., 2022). These previous findings are sufficient to indicate high involvement of PHF14 in various disease development and progression, including oncogenesis.

The international public repository GEO and the publicly funded project TCGA, which contains numerous publicly available cancer genome datasets, aim to classify and discover primary oncogenic genomic alterations through large-scale genome sequencing and comprehensive multidimensional analysis, thereby creating a comprehensive Cancer Genome Profile “atlas” (Barrett et al., 2013; Zhang et al., 2019). In recent years, individual studies and pan-cancer analysis of cancer have provided fresh ideas for tumor diagnosis and treatment. Although the strong correlation between PHF14 and individual tumors has been gradually identified, how PHF14 expression interacts with carcinogenesis and clinical prognosis of multiple tumor types has not been comprehensively evaluated through pan-cancer analysis. Through the GEO database and TCGA project, this paper will provide the first PHF14 (NP_055475.2 for protein or NM_014660 for mRNA) pan-cancer analysis to systematically describe the differential expression, genetic alterations, immune infiltration, related gene enrichment analysis of PHF14 and survival prognosis among different cancer types. In conclusion, this study provides new ideas to study the pathogenesis or clinical prognosis of human PHF14 in multiple tumors.

## Materials and methodology

### PHF14 gene expression analysis

The data of clinical profiles and gene expression matrix of each normal and tumor individuals in TCGA database and GTEx database were processed from UCSC XENA database (https://xenabrowser.net/datapages/). Thereinto, the gene expression of 33 cases of cancer was analyzed through integration of normal tissue data from the GTEx database and tumor tissue data from the TCGA database. Moreover, 18 tumors and paired adjacent non-cancerous tissues were taken from the TCGA database for gene expression analysis. Data on pan-cancer immune infiltrating cells scores were obtained from the timer database (https://cistrome.shinyapps.io/timer/) and data on the expression of each tumor cell line were downloaded from CCLE database (https://portals.broadinstitute.org/). A PHF14 mRNA expression plot was constructed in tissue and cells with the Human Protein Atlas (HPA) database (version: 20.1) (https://www.proteinatlas.org/).

An edgeR software was adopted to analyze the differences in PHF14 expression levels in normal tissues, tumor tissues and paired non-cancerous tissues. Wilcoxon rank sum test was applied to analyze PHF14 expression level in different normal tissues and different tumor cell lines. Box plots were plotted by R package ggplot. UCSC XENA database (https://xenabrowser.net/datapages/) contributed clinicopathological features and RNA-sequencing (RNA-seq) data of these 33 cancers, namely, adrenocortical carcinoma (ACC), bladder urothelial carcinoma (BLCA), breast invasive carcinoma (BRCA), cervical squamous cell carcinoma (CESC), cholangiocarcinoma (CHOL), colon adenocarcinoma (COAD), lymphoid neoplasm diffuse large B cell lymphoma (DLBC), esophageal carcinoma (ESCA), glioblastoma (GBM), brain lower grade glioma (LGG), head and neck squamous cell carcinoma (HNSC), kidney chromophobe (KICH), kidney renal clear cell carcinoma (KIRC), kidney renal papillary cell carcinoma (KIRP), acute myeloid leukemia (LAML), liver hepatocellular carcinoma (LIHC), lung adenocarcinoma (LUAD), lung squamous cell carcinoma (LUSC), mesothelioma (MESO), ovarian serous cystadenocarcinoma (OV), pancreatic adenocarcinoma (PAAD), pheochromocytoma and paraganglioma (PCPG), prostate adenocarcinoma (PRAD), rectum adenocarcinoma (READ), sarcoma (SARC), skin cutaneous melanoma (SKCM), stomach adenocarcinoma (STAD), testicular germ cell tumors (TGCT), thyroid carcinoma (THCA), thymoma (THYM), uterine corpus endometrial carcinoma (UCEC), uterine carcinosarcoma (UCS) and uveal melanoma (UVM). Log2 conversion was performed on all expression data. Nevertheless, MESO and uveal UVM have no normal or highly normal tissue deficiency in this module.

PHF14 was entered into the “CPTAC (Clinical proteomic tumor analysis consortium) analysis” module with the permission of the UALCAN portal (http://ualcan.path.uab.edu/analysis-prot.html), and the total protein expression level of PHF14 between normal tissues and primary tumors of TCGA project were compared. There were 10 tumor datasets identified in this study (breast cancer, clear cell RCC, colon cancer, GBM, HNSC, LIHC, LUAD, ovarian cancer, PAAD and UCEC). Finally, PHF14 expression violin plots was determined by the ‘Pathological Stage Plot’ module of GEPIA2 for different pathological stages (stage I, II, III, IV and V) of different tumors in TCGA. The violin plots were converted into forlog2 [Transcripts per million (TPM) +1] expression data.

### Survival analysis

To further explore how gene expression of PHF14 affects all TCGA tumors’ survival and prognosis, the “Survival Analysis” module of the Gene Expression Profile Interactive Analysis version 2 (GEPIA2) (http://gepia2.cancer-pku.cn/) was applied to generate the overall survival (OS) and disease-free survival (DFS) and Kaplan–Meier (K-M) plots of PHF14 across all tumors from the TCGA database. Also, UCSC Xena Browser was adopted to analyze the progression-free survival (PFS) of the TCGA Pan-Cancer datasets (version: 2018–09–13) (https://xenabrowser.net/) (settings: cutoff-low: 50% cutoff-high value: 50%). Hypothesis test was performed using log-rank tests.

### Genetic alteration analysis

In this study, PHF14 genetic alterations in cancer were studied with cBioPortal (version: 3.6.20) (https://www.cbioportal.org/). We input “PHF14” in the Quick Selection section of the cBioPortal web to explore the genetic alteration characteristics of PHF14 in TCGA Pan Cancer Atlas Studies. Next, a mutation site plot of PHF14 was generated by using the “Mutations” module, including the alteration frequency results, CNA (Copy number alteration), mutation type, and structural variants. Finally, the differences in the overall survival as well as disease-free and progression-free survival among UCEC cancer patients with or without PHF14 genetic alteration were analyzed using the “comparison/survival” module. Survival data were visualized using Kaplan-Meier curves.

### Immune infiltration analysis

In this study, PHF14 expression in tumor tissue and adjacent normal tissue of different types of cancers was examined with the “Immune-Gene” module of Tumor Immune Estimation Resource version 2 (TIMER2) (http://timer.cistrome.org/) was adopted to examine PHF14 expression in tumor tissues of various type of cancers and adjacent normal tissues in the TCGA project and also, the immune cells of cancer-associated fibroblasts (CAFs) were included for analysis. Subsequently, the degree of tumor immune infiltration was assessed with part or all of the EPIC, MCPCOUNTER, and TIDE algorithms. After purity adjustment, the P-values were determined through Spearman rank correlation test. Data were visualized using heatmaps and scatterplots.

### Association analysis of PHF14 with immune checkpoints genes

To analyze how PHF14 and immune checkpoint genes correlate with each other, data of 33 normal and tumor tissues in the TCGA database were obtained from the Genomic Data Commons (GDC) data portal. These immune checkpoint genes were extracted and their correlation with PHF14 expression was calculated through analysis of Spearman correlation analysis heat maps between multiple tumor immune checkpoint genes and PHF14.

## Analysis of the association of PHF14 with DNA mismatch repair genes and methyltransferases

Mismatch repair an intracellular mismatch repair mechanism that involves the loss of key gene function leading to irreparable DNA replication errorsand subsequent induction of elevated levels of mutations into somatic cells. This study used the expression profiling data from the TCGA database to determine the correlation between 5 MMRs genes (EPCAM, MLH1, MSH2, MSH6 and PMS2) and PHF14 expression. DNA methylation, a chemically modified form on DNA, can modify epigenetic inheritance and control gene expression with no modifications to the DNA sequence. Herein, an analysis of PHF14 expression and the associated expression of three methyltransferases (including DNMT1, DNMT3A and DNMT3B) were made using the TIMER2 “Gene_Corr” module. The heat map shows Spearman’s rank correlation test P-value and purity adjusted partial correlation (COR) value. When *p* < 0.05 and R > 0.20, the correlation was considered significant and positive.

### PHF14 co-expressed gene enrichment analysis

The STRING tool (https://string-db.org/) was adopted to construct a *Homo Sapiens* PHF14 co-expression network. At the same time, meet the following parameter requirements: 1) Fill in “50” in the maximum number of interactors; 2) Fill in “low confidence (0.150)” in the minimum required interaction score; 3) Fill in “co-expression” in the active interaction sources; and 4) Fill in “evidence” in the meaning of network edge. Finally, a co-expression network of 50 genes co-expressed with PHF14 was yielded. Subsequently, the visualization of the PPI network was realized by Cytoscape software. Furthermore, a combination of 50 genes co-expressed with PHF14 was enriched for KEGG (Kyoto Encyclopedia of Genes and Genomes) and GO (Gene Ontology) pathway analysis with the ClusterProfiler package to gain further insight into the biology and molecular features of these genes.

Gene set enrichment analysis (GSEA) is commonly used as an analytical method in comparing the expression status of genes and predefined gene sets with a particular biological process, cellular component or molecular function, so as to explore whether they are statistically significant to a certain extent. The threshold for GSEA was set at |NES| > 1, *p*-value <0.05, FDR <0.25, and if pathways met the sub-conditions, it was assumed that they are significantly enriched.

### Human protein atlas

The Human Protein Atlas (https://www.proteinatlas.org) is a website that contains immunohistochemistry-based expression data for near 20 highly common kinds of cancers (Asplund et al., 2012). The user can identify the tumor types of specific protein expression patterns, these proteins are differentially expressed in certain types of cancer. In this study, the protein expression of PHF14 in human normal and KIRC tissues, human normal and PAAD tissues, and human normal and LIHC tissues were directly compared using immunohistochemical images from this website.

### Cell and cell culture

The human kidney renal clear cell carcinoma cell lines Caki-2 and 786-O, human pancreatic duct epithelial cell line hTERT-HPNE, human pancreatic cancer cell lines ASPC-1 and PANC-1, and human liver hepatocellular carcinoma cell lines MHCC-97h and Huh-7 were preserved and cultured in Dulbecco’s m o d ified Eagle’s medium (DMEM)-high glucose (Gibco, United States) containing 10% Fetal Bovine Serum (FBS, Gibco, United States) and 1% penicillin/streptomycin. The both human renal tubular epithelial cell line HK-2 and human normal liver cell line LO2 were preserved and cultured in RPMI-1640 (Gibco, United States) containing 10% FBS and 1% penicillin/streptomycin. Before the following experiments, all cells were kept under standard adherent conditions of 37°C, 5% CO_2_ and humidified atmosphere.

### Real-time PCR analysis

All cells (2 × 10^–6^ cells/well) were inoculated into 6-well plates and cultured for 48 h. Total RNA was isolated using TRIzol reagent (Invitrogen Carlsbad, CA, United States). For RT-PCR, single-stranded cDNA was synthesized from 1 µg of total RNA using reverse transcriptase (TaKaRa Biotechnology, Otsu, Japan). In addition, primers were designed against the following human sequences: PHF14(forward:AGTGCTCGGAATGTGACCAG,reverse:CCATCCGTAGCCTGTCTGTT),GAPDH (forward:GGAAGCTTGTCATCAATGGAAATC,reverse:TGATGACCCTTTTGGCTCCC). Real-time PCR was performed using SYBR^®^ Premix ExTaq™ II (Tli RNaseH Plus) (TaKaRa Biotechnology) and results were detected using an ABI 7500 Sequencing Detection System (Applied Biosystems, Foster City, CA, United States). The thermal cycling conditions were 95°C for 30 s and then 40 cycles of 95°C for 5 s and 60°C for 34 s.

### siRNA-mediated knockdown

PHF14-specific siRNA were purchased from GenePharma (Shanghai, China). The corresponding target sequence of siRNA are shown as following: forward: CCA​GUA​ACA​CUA​ACG​GAA​ATT, reverse: UUU​CCG​UUA​GUG​UUA​CUG​GTT. PHF14-specific siRNA was represented by siPHF14 and negative control group by siNC. Lipofectamine 2000 reagent (Cat. No. 11668019, Invitrogen) was used to deliver siRNA and siNC into the human liver hepatocellular carcinoma cell lines MHCC-97h and human pancreatic cancer cell lines PANC-1. The final concentration of 20 μM siRNA and 1 mg/mL Lipofectamine 2000 reagent were diluted with OptiMEM (Gibco) before transfection. The culture medium was changed into DMEM containing 10% FBS and 1% penicillin/streptomycin after 4–6 h in carbon dioxide incubator at 37°C. Two days or 3 days after transfection, the expression level of PHF14 was detected by qRT-PCR to assess the validation of the knockdown. According to the manufacturer’s instructions, the cell viability was assessed by measuring 3-(4,5-dimethylthiazol-2-yl)-2,5-di-phenyltetrazolium bromide (Nacalai Tesque, Kyoto, Japan) dye absorbance (MTT assay) at 1,2, 3, and 4 days after siRNA transfection.

### EdU assays

EdU Apollo 567 Cell Tracking Kit (Rib-bio, Guangzhou, China, Cat# C10310-1) was used to evaluate the proliferation of MHCC-97h and PANC-1 cells. PHF14 knock-down cells and negative control cells (1 × 10^4^/well) were inoculated in 96-well plates and incubated overnight at 37°C. Then 5-ethynyl-20-deoxyuridine (EdU, 200 μM) was added and incubated for 2 h at 37°C. Cells were cold fixed with 4% paraformaldehyde for 20 min, washed three times with PBS, and then treated with 0.5% Triton X-100 at room temperature for 10 min. Then, after washing with PBS for 3 times, each well was incubated with 100 μL Apollo reagent for 30 min. Finally, the nuclei were stained with Hoechest 33,342 for 5 min. The percentage of EdU-positive cells was calculated based on counts from 500 cells in three independent experiments.

### Invasion and migration assays

The invasion potential of PANC-1 and MHCC-97h cells was evaluated by Transwell-Matrigel system. The culture upper inserts were coated with Matrigel (BD Matrigel, United States, Cat# 356234). The cells were resuspended in serum-free DMEM medium, and the cell density was adjusted to 1-10*10^5^/mL. 200μL cell suspension was added to each transwell upper chamber (24 wells, 8 mm pore size; BD Biosciences, United States, Cat# 3428), and DMEM medium containing 10%FBS was added to each lower chamber. After incubation for 48 h, the cells and Matrigel in the upper chamber were removed with a cotton swab and the cells adhering to the lower membrane of the inserts were fixed in ice-cold methanol at 4°C and stained with 1% crystal violet. Quantification of cell invasion was expressed as the mean count of stained cells in 5 random fields of each membrane under light microscope (×20objective lens). The migration ability of PANC-1 and MHCC-97h cells was analyzed by cell scratch assay. The cells were cultured in serum-free DMEM medium and performed scratch assay with pipette tip. The cell migration was compared and observed after 6, 24, and 48 h. All the experiments were performed in triplicates.

### Cell apoptosis measured by flow cytometry

An annexin V-fluorescein isothiocyanate (FITC) apoptosis detection kit (BD Biosciences, San Jose, CA, United States) was used to measure apoptotic cells. PANC-1 and MHCC-97h cells were treated with siNC(Negative control-lentivirus) and siPHF14 (PHF14-siRNA-lentivirus) for 48 h, then collected and resuspended with 500 μL 1X binding buffer. Then, 5 μL of annexin V-FITC and 10 μL of propidium iodide (PI) were added. After gentle mixing, incubate at room temperature and away from light for 5 min. Finally, the stained cells were analyzed using a flow cytometer (BD Biosciences, San Jose, CA, United States).

## Results

### PHF14 expression in pan-cancer

**FIGURE 1:**
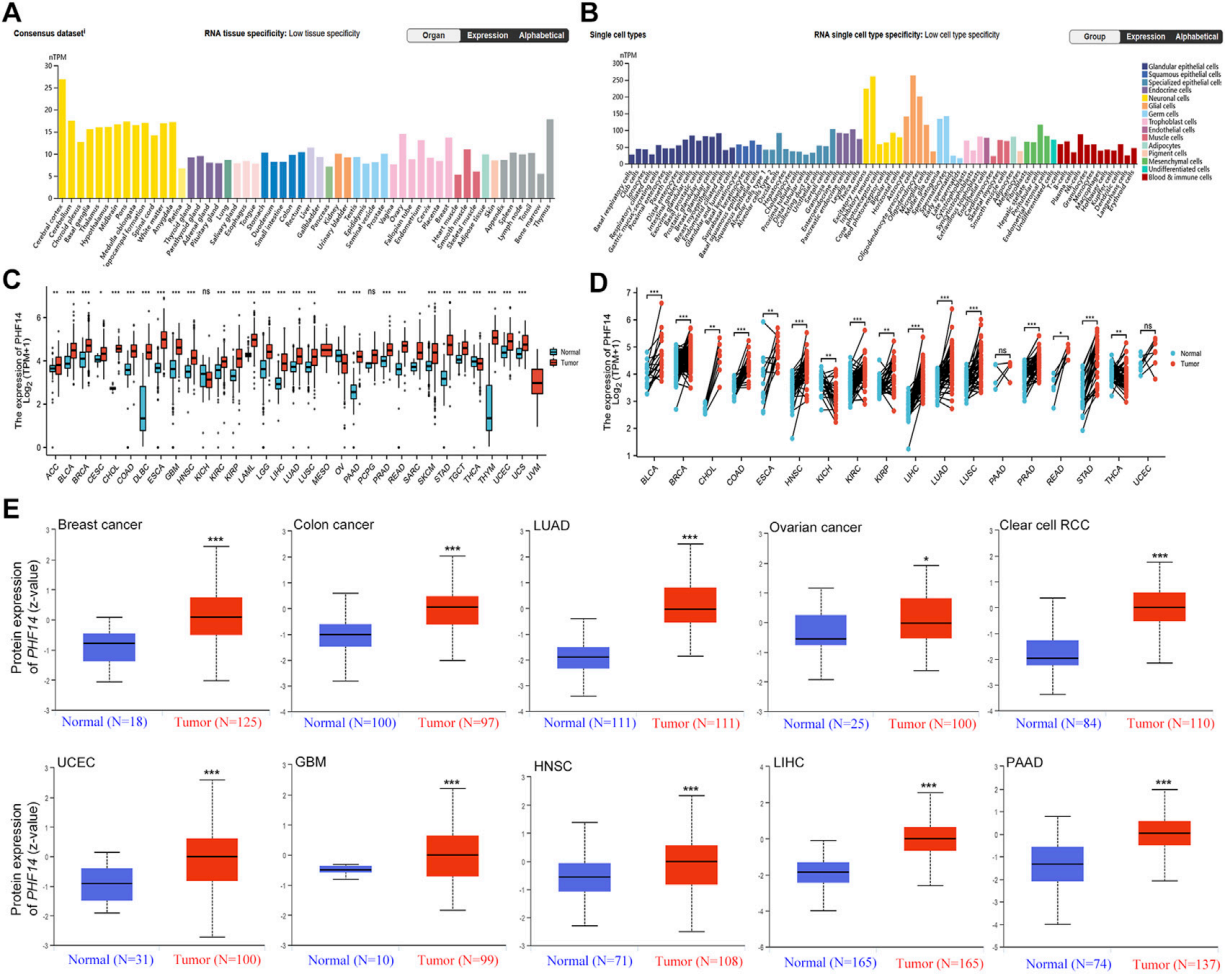
The PHF14 expression status in different tumors and normal tissues. **(A)** Consensus PHF14 tissue expression based on datasets of HPA (Human Protein Atlas), GTEx, and FANTOM5 (function annotation of the mammalian genome). **(B)** Consensus PHF14 cell type expression based on the above datasets. **(C,D)** The TCGA project’s PHF14 gene expression difference in different tumors or specific tumor subtype tissues and unpaired or paired adjacent normal tissues was analyzed by TIMER2. *p < 0.05; **p < 0.01; ***p < 0.001. **(E)** Difference of the PHF14 total protein expression between normal and tumor tissues of breast cancer, colon cancer, LUAD, clear cell RCC, UCEC, GBM, HNSC, LIHC, PAAD and ovarian cancer were analyzed based on the CPTAC dataset. *p < 0.05; ***p < 0.001.

Using HPA, GTEx, and FANTOM5 (Functional Annotation of Mammalian Genomes) datasets, PHF14 was shown to be enriched in thymus and ovary, and highly expressed in cerebral cortex and cerebellum among other cancers (Figure 1A; Supplementary Figure S1). In addition, PHF14 was also highly expressed in neuronal cells and glial cells based on single-cell RNA-seq (Figure 1B). According to our data, we found a lower PHF14 expression in terms of its tissue-specific and cell type specificity.

From an unpaired perspective (Figure 1C), significant increases in PHF14 expression levels were observed in tumor specimen tissues of adrenocortical carcinoma (ACC) (*p <* 0.01), cervical squamous cell carcinoma (CESC) (*p <* 0.05), bladder urothelial carcinoma (BLCA), breast invasive carcinoma (BRCA), cholangiocarcinoma (CHOL), colon adenocarcinoma (COAD), lymphoid neoplasm diffuse large B cell lymphoma (DLBC), esophageal carcinoma (ESCA), glioblastoma (GBM), head and neck squamous cell carcinoma (HNSC), kidney renal clear cell carcinoma (KIRC), kidney renal papillary cell carcinoma (KIRP), acute myeloid leukemia (LAML), brain lower grade glioma (LGG), liver hepatocellular carcinoma (LIHC), lung adenocarcinoma (LUAD), lung squamous cell carcinoma (LUSC), pancreatic adenocarcinoma (PAAD), prostate adenocarcinoma (PRAD), rectum adenocarcinoma (READ), skin cutaneous melanoma (SKCM), stomach adenocarcinoma (STAD), testicular germ cell tumors (TGCT), thymoma (THYM), uterine corpus endometrial carcinoma (UCEC) and uterine carcinosarcoma (UCS) as compared to adjacent normal tissues (*p <* 0.001). However, PHF14 expression levels was lower in ovarian serous cystadenocarcinoma (OV) and thyroid carcinoma (THCA) tumor tissues than in adjacent normal tissues (*p < 0.001*). Moreover, PHF14 expression levels in kidney chromophobe (KICH) and pheochromocytoma and paraganglioma (PCPG) tumor tissues were also not distinctly different from those of the adjacent normal tissues (ns: *p >* 0.05). From a pairing point of view (Figure 1D), compared with the adjacent normal tissues, PHF14 expression levels were elevated markedly in the tumor tissues of bladder urothelial carcinoma (BLCA), breast invasive carcinoma (BRCA), colon adenocarcinoma (COAD), head and neck squamous cell carcinoma (HNSC), kidney renal clear cell carcinoma (KIRC), liver hepatocellular carcinoma (LIHC), lung adenocarcinoma (LUAD), lung squamous cell carcinoma (LUSC), prostate adenocarcinoma (PRAD), stomach adenocarcinoma (STAD) (*p < 0.001*), cholangiocarcinoma (CHOL), kidney renal papillary cell carcinoma (KIRP), thyroid carcinoma (THCA) (*p < 0.01*) and rectum adenocarcinoma (READ) (*p < 0.05*). However, higher levels of PHF14 expression were observed in adjacent normal tissues than that in esophageal carcinoma (ESCA) and kidney chromophobe (KICH) tumor tissues (*p <* 0.01). It was also observed that PHF14 expression levels was not statistically significant between the adjacent normal tissues and the pancreatic adenocarcinoma (PAAD) and uterine corpus endometrial carcinoma (UCEC) tumor tissues.

**FIGURE 2:**
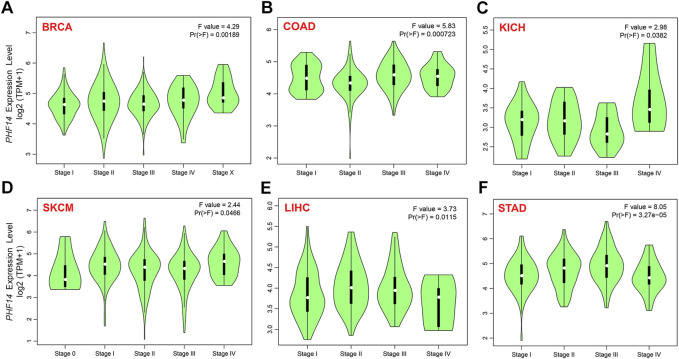
On the basis of the TCGA dataset, **(A–F)** analysis of the expression level of PHF14 gene in the different pathological stages (stage I, II, III, and IV) in BRCA, COAD, KICH, SKCM, LIHC and STAD tumors by applying GEPIA2.

To clarify PH14 protein expression levels in various tumors, CPTAC dataset protein expression was analyzed. In Figure 1E, compared with adjacent normal tissues, the total protein expression level of PHF14 was higher in primary tumor tissues of breast cancer, colon cancer, kidney renal clear cell carcinoma (clear cell RCC), lung adenocarcinoma (LUAD), uterine corpus endometrial carcinoma (UCEC), glioblastoma (GBM), head and neck squamous cell carcinoma (HNSC), liver hepatocellular carcinoma (LIHC), pancreatic adenocarcinoma (PAAD) (*p < 0.001*) and ovarian cancer (*p < 0.05*). In addition, the relationship between PHF14 expression level and different tumor pathological stages was investigated using the GEPIA2. In Figures 2A–F, PHF14 expression levels in BRCA, COAD, KICH, SKCM, LIHC and STAD tumors at different pathological stages were significantly different (*p* < 0.05).

### Association between PHF14 expression and survival prognosis

**FIGURE 3:**
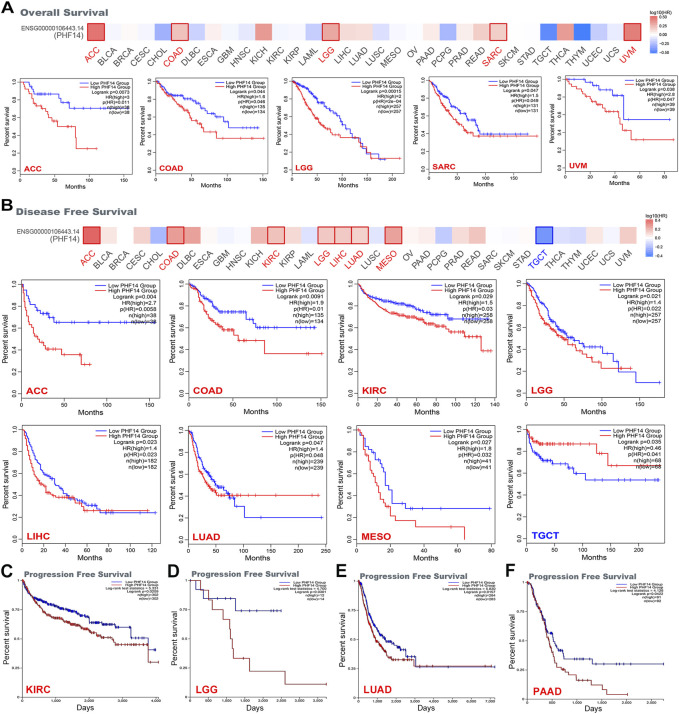
Correlation between PHF14 gene expression and survival prognosis for tumors in TCGA analyzed with the GEPIA2 tool. **(A)** Overall survival analysis. **(B)** Disease-free survival. **(C–F)** Progression-free survival in KIRC, LGG, LUAD, and PAAD. The results with significant differences were visualized through a survival map and Kaplan-Meier curves.

The cancer cases included in this study were sorted into a low PHF14 expression group and a high PHF14 expression group based on PHF14 expression. The survival prognosis between the two groups in different cancers was assessed and compared using the TCGA and GEO datasets. As can be seen from Figure 3A, high PHF14 expression was relevant to poor OS (overall survival) prognosis of patients with ACC (*p* = 0.0073), COAD (*p* = 0.044), LGG (*p* = 0.00015), SARC (*p* = 0.047) and UVM(*p* = 0.038).

Likewise, in Figure 3B, high PHF14 expression was correlated to inferior prognosis of patients with ACC (*p* = 0.004), COAD (*p* = 0.0091), KIRC (*p* = 0.029), LGG (*p* = 0.021), LIHC (*p* = 0.023), LUAD (*p* = 0.047) and MESO (*p* = 0.027) from DFS (disease-free survival) analysis. However, low PHF14 gene expression was relevant to poor OS prognosis in TGCT patients (*p* = 0.035). Moreover, as indicated in Figures 3C–F, high PHF14 expression was relevant to poor progression-free survival in patients with KIRC (*p* = 0.0209), LGG (*p* = 0.0301), LUAD (*p* = 0.0157), and PAAD (*p* = 0.0422). From the above results, it can be observed that PHF14 expression levels are relevant to the prognosis of patients with pan-cancer, and variations can be observed in accordance with the type of cancer.

### PHF14 genetic alteration of PHF14 in various tumors

**FIGURE 4:**
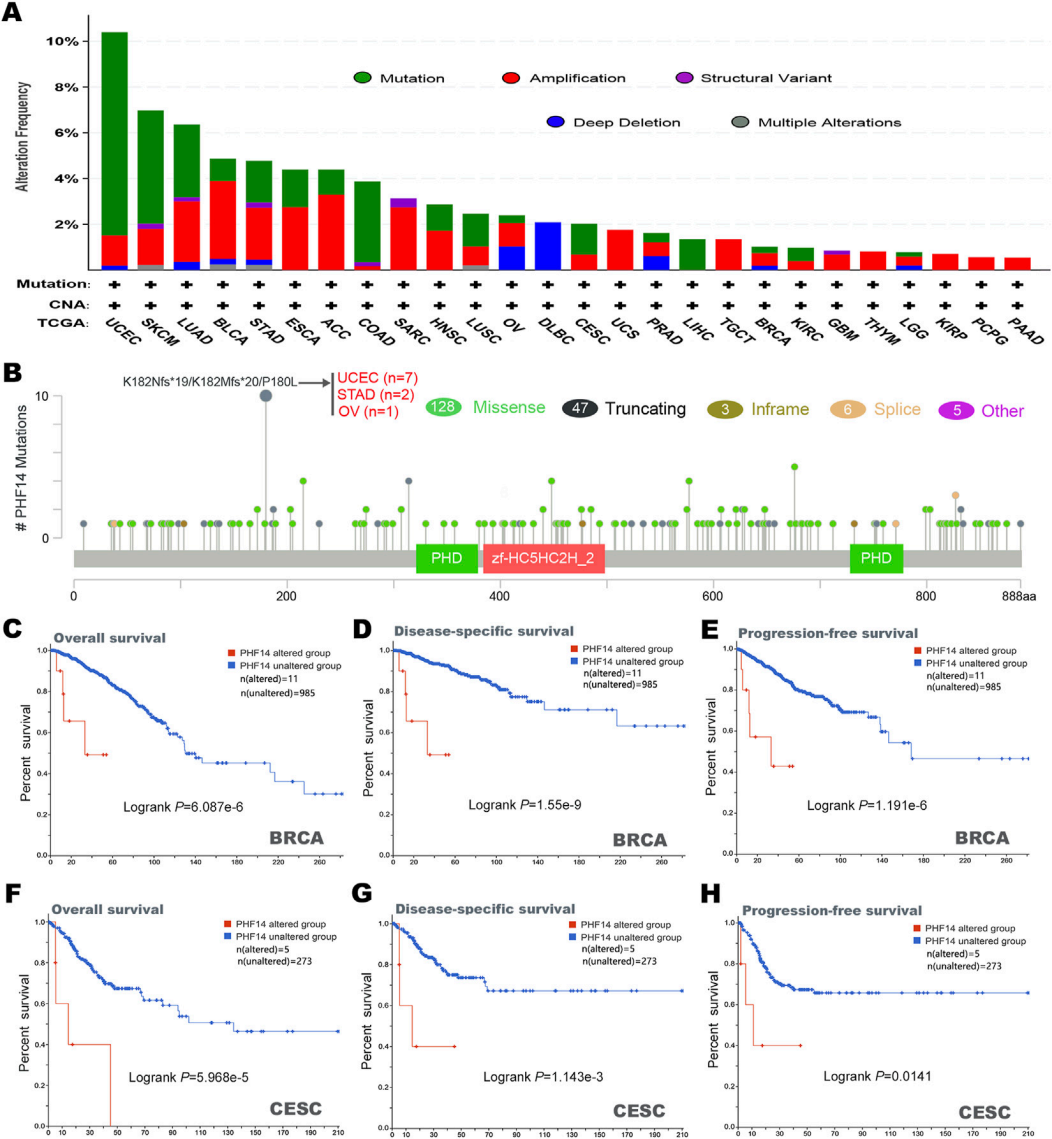
Mutation characteristics of PHF14 gene in different kind of tumors of TCGA were analyzed by the cBioPortal tool. **(A)** The mutation type and alteration frequency in various cancers. **(B)** The mutation sites in PHF14. **(C–H)** The correlation between MSH6 mutation status and overall, disease-specific, and progression-free survival prognoses of BRCA and CESC.

In this study, cBioPortal was adopted to investigate the genetic changes of PHF14 in various types of tumors in the TCGA dataset. It was demonstrated in Figure 4A that the highest alteration frequency (>10%) of the PHF14 gene appeared in UCEC tumor samples, predominantly expressed in the “mutation” type (>8%). In addition, the “mutation” type was the predominant type of genetic alteration in SKCM patients, accounting for approximately 4%. Notably, all types of PHF14 gene alterations (∼2% frequency) in LIHC patients were “mutation.” Moreover, all alterations of PHF14 gene in UCS, TGCT, THYM, KIRP, PCPG and PAAD patients were “amplification,” with this being the predominant type of genetic alteration in all TCGA tumor samples.

In Figure 4B, we can further observe the type, location and number of PHF14 genetic alterations. In addition, we also detected alterations in the K182Nfs*19/K182Mfs*20/P180L in two cases of STAD, seven cases of UCEC and one case of OV, and the major type of PHF14 gene mutation was missense mutation. Subsequently, how PHF14 gene mutation reacted with survival prognosis of patients with various types of tumors was examined in this study. As shown in Figures 4C–H, in BRCA and CESC cancer patients, the PHF14-unaltered group exhibited a favourable prognosis for DSS (disease-specific survival) (P-values were 1.55e-9 and 1.143e-3, respectively), OS (P-values were 6.087e-6 and 5.968e-5, respectively) and PFS (P-values were 1.191e-6 and 0.0141, respectively) as compared to the PHF14-altered group. The above results indicate that PHF14 expression in pan-cancer is relevant to PHF14 amplification and mutation, further suggesting that the genetic alterations of PFH14 are highly relevant to survival prognosis of different cancer patients.

### Cancer-associated fibroblast immune infiltration analysis

**FIGURE 5:**
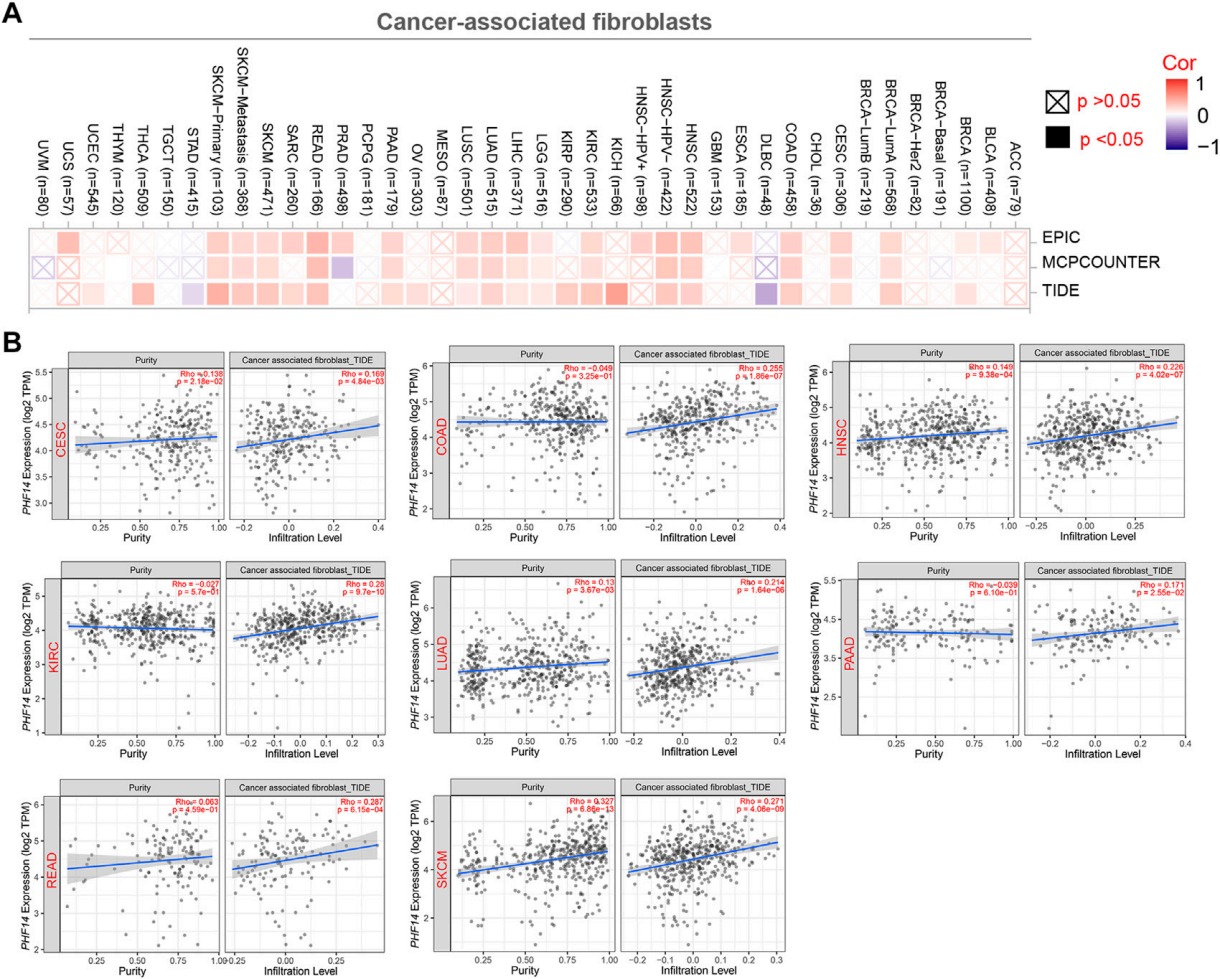
Correlation analysis between PHF14 expression and immune infiltration of cancer-associated fibroblasts. **(A)** The correlation between PHF14 expression and immune infiltration of cancer-associated fibroblasts for all TCGA tumors evaluated by different algorithms, including EPIC, MCPCOUNTER and TIDE. **(B)** Scatter plot data for selected tumors generated using one of the algorithms were provided.

Figure 5 presents how infiltration of cancer-associated fibroblasts (CAFs) interacts with PHF14 gene expression in different types of cancers in TCGA. A positive correlation between PHF14 expression and the level of infiltration of cancer-associated fibroblasts in CESC, COAD, HNSC, KIRC, LUAD, PAAD, READ and SKCM can be observed using the EPIC, MCPCOUNTER and TIDE algorithms. Additionally, TIDE algorithm was used to generate the above-mentioned tumor scatter plot data, as indicated in Figure 5B. The findings reveal that cancer-associated fibroblast immune infiltration is closely correlated with cancer occurrence, development or metastasis.

### Correlation analysis of PHF14 expression and immune checkpoint genes in pan-cancer

**FIGURE 6:**
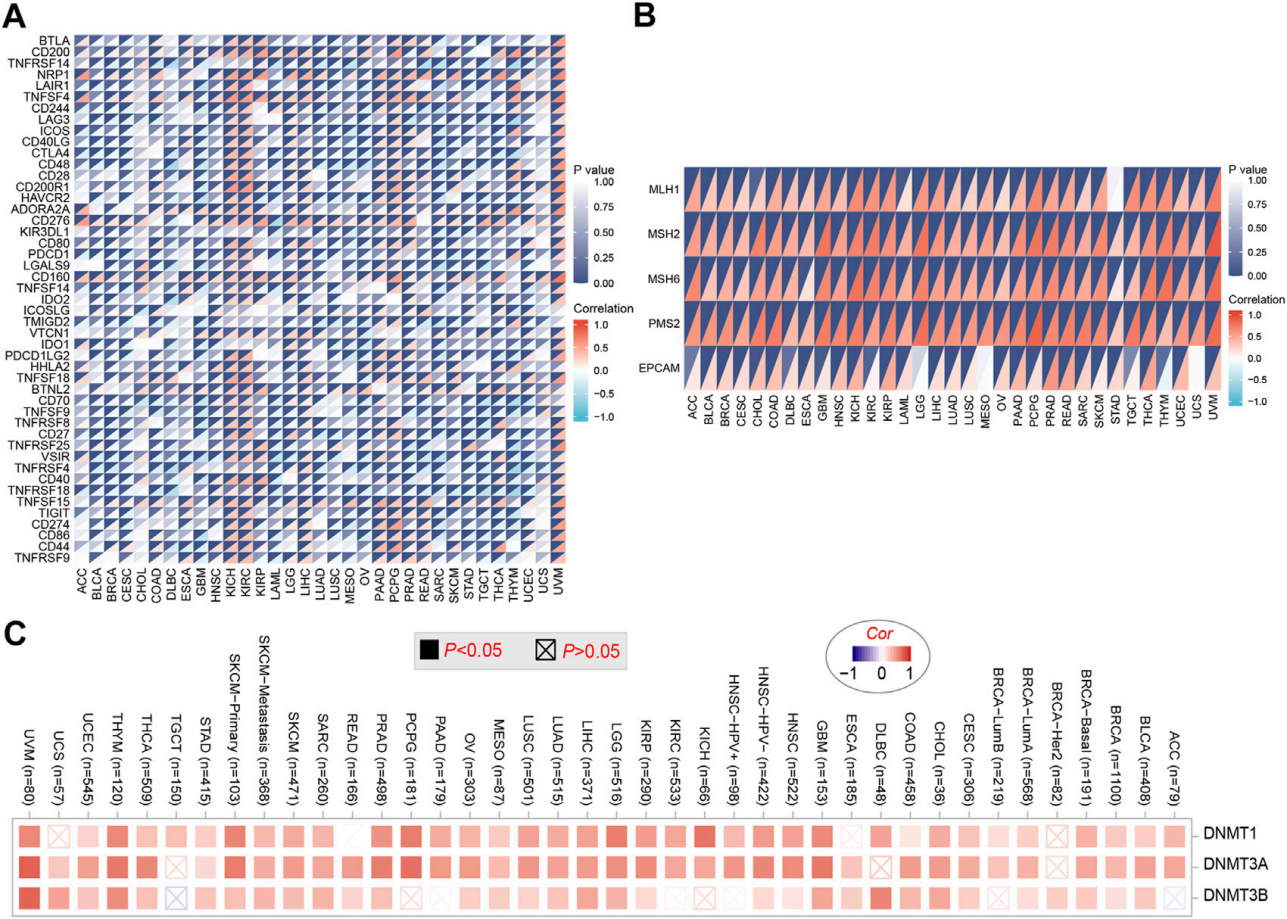
Correlation analysis of PHF14 expression and immune checkpoint genes in pan-cancer. **(A)** Correlation analysis between PHF14 expression in Pan-cancer and immune checkpoint gene expression. **(B)** Correlation analysis of PFH14 expression and the expression levels of five common MMRs genes (MLH1, MSH2, MSH6, PMS2, EPCAM) in various types of tumors in TCGA. **(C)** Corresponding heatmap data for targeted genes (DNMT1, DNMT3A, DNMT3B) in selected cancer types in TCGA.

To explore the relationship between PHF14 expression and immune checkpoint gene expression in this study, an analysis of the expression data of 47 immune checkpoint genes commonly found in various tumors was performed and the findings are presented in Figure 6. As revealed in Figure 6A, PHF14 expression is in positive correlation with the expression levels of almost all immune checkpoint genes in KICH, KIRC, UVM and other tumors. The above results indicate that in some tumors, the regulation of immune checkpoint gene expression levels by PHF14 may affect tumor immunity.

### PHF14 affects DNA mismatch repair genes and methyltransferas expression in pan-cancer

As demonstrated in Figure 6B, we can find that the 5 common MMRs genes (EPCAM, MLH1, MSH2, MSH6 and PMS2) exhibited highly positive association with PHF14 expression in various types of TCGA tumors, suggesting that PHF14 could maintain the viability of tumor cells by up-regulating genes related to DNA mismatch repair. In addition, DNA methylation was produced as a result of DNA methyltransferases, which covalently binds a methyl group at the 5’ carbon position of cytosine, a CpG dinucleotide in the genome. As shown in Figure 6C, the heatmap data obtained from the TIMER2 online tool revealed that PHF14 and methyltransferase expression levels positively correlated with most TCGA tumor types, suggesting that PHF14 can mediate tumorigenesis and progression through regulating human pan-cancer epigenetic status.

### Function enrichment analysis of PHF14 co-expressed gene

**FIGURE 7:**
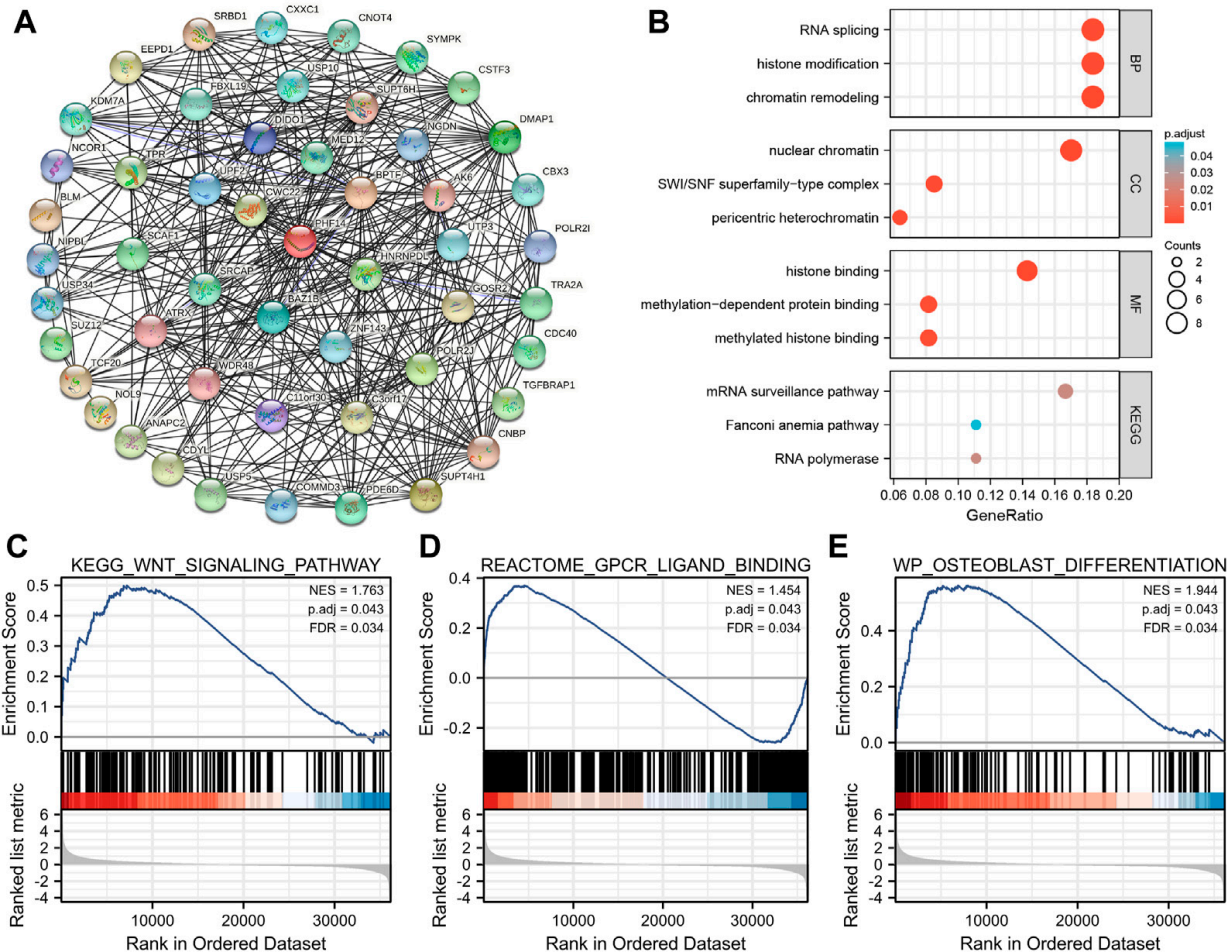
PHF14-related gene function enrichment analysis. **(A)** On the basis of the STRING tool, Co-expression network of 50 genes co-expressed with PHF14 were obtained. **(B)** CC, BP, MF and KEGG pathways analysis based on PHF14-correlated genes and PHF14-binding protein. **(C–E)** Results of GSEA of the top 3 rankings of PHF14 correlation with signaling pathways in KEGG database.

In this study, PHF14 co-expressed genes were analyzed for pathway enrichment to explore the mechanism of PHF14 gene in tumor. An experimentally validated set of 50 available msh6 binding proteins was generated by using the STRING tool. The interaction network of these PHF14-binding proteins is clearly presented in Figure 7A. Furthermore, in Figure 7B, we combined 50 PHF14 co-expressed genes for CC, BP, MF and KEGG enrichment analysis. Enrichment analysis of KEGG pathway revealed that “mRNA surveillance pathway” may be involved in the pathogenesis of tumor PHF14. The results of MF and CC showed that “histone binding” and “nuclear chromatin” may be involved in the tumorigenesis and development mechanism of PHF14. Finally, the enrichment results of BP pathway revealed that, “RNA splicing,” “histone modification” and “chromatin remodeling” may all be involved in the pathogenesis of tumor PHF14. Furthermore, based on PHF14 expression levels, human pan-cancer samples were divided into high and low expression groups, and the enrichment of signaling pathways in KEGG and the markers of the two groups were analyzed by GSEA. As shown in Figures 7C–E, we have listed the top 3 most significantly enriched signaling pathways in the KEGG database, namely, KEGG_WNT_SIGNALING_PATHWAY (NES = 1.763; P. adjust = 0.043; FDR = 0.034), REACTOME_GPCR_LIGAND_BINDING (NES = 1.454; P. adjust = 0.043; FDR = 0.034), and WP_OSTEOBLAST_DIFFERENTIATION (NES = 1.944; P. adjust = 0.043; FDR = 0.034), which suggested that PHF14 may be involved in signaling pathways regulating tumor metabolism and immunity.

### Protein expression and transcription of PHF14 in patients

**FIGURE 8:**
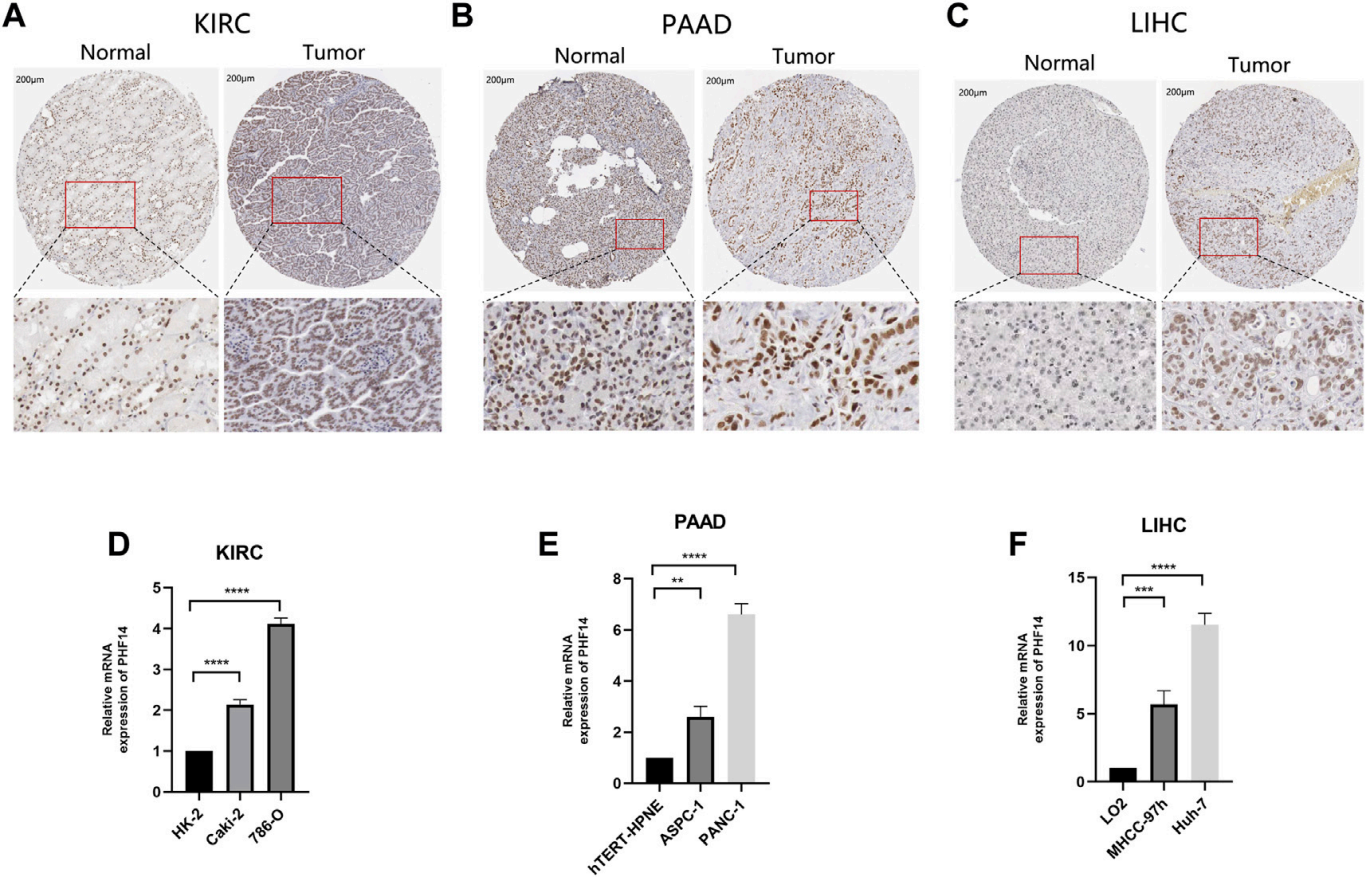
Validation of PHF14 gene at translational and transcriptional levels. **(A–C)** Immunohistochemistry images from The Human Protein Atlas database were used to verify the translation expression level of PHF14 gene in KIRC, PAAD, and LIHC. The figures showed that PHF14 protein was highly expressed in the above three kinds of tumor tissues compared with the corresponding normal tissues. **(D–F)** Real-time PCR analysis of PHF14 transcription levels in KIRC, PAAD and LIHC showed that the mRNA expression level of PHF14 in human kidney renal clear cell carcinoma cell lines (Caki-2 and 786-O) was higher than that in human renal tubular epithelial cell line (HK-2). And the mRNA expression of PHF14 in human pancreatic adenocarcinoma cell lines (ASPC-1 and PANC-1) and human liver hepatocellular carcinoma cell lines (MHCC-97h and Huh-7) was higher than that of human pancreatic duct epithelial cell line (hTERT-HPNE) and human normal liver cell line (LO2), respectively. **p < 0.01; ***p < 0.001; ****p < 0.0001.

We tried to explore the protein expression patterns of PHF14 in KIRC, PAAD, and LIHC by the Human Protein Atlas. As shown in Figures 8A–C, PHF14 protein were highly expressed in tumor tissues (KIRC, PAAD, and LIHC) compared to normal tissues. After finding the difference in the expression of PHF14 protein in normal and tumor tissues, we cultured tumor cell lines and corresponding normal cell lines *in vitro*. As shown in Figures 8D–F, PCR results showed that the mRNA expression level of PHF14 in human kidney renal clear cell carcinoma cell lines (Caki-2 and 786-O) was higher than that in human renal tubular epithelial cell line (HK-2). Similarly, The mRNA expression of PHF14 in human pancreatic adenocarcinoma cell lines (ASPC-1 and PANC-1) and human liver hepatocellular carcinoma cell lines (MHCC-97h and Huh-7) was higher than that of human pancreatic duct epithelial cell line (hTERT-HPNE) and human normal liver cell line (LO2), respectively. Taken together, our results showed that transcriptional and proteinic expressions of PHF14 were over-expressed in patients with KIRC, PAAD and LIHC.

### PHF14-knockdown inhibited the growth, migration and invasion of PANC-1 and MHCC-97h cells, and promoted cells apoptosis

**FIGURE 9:**
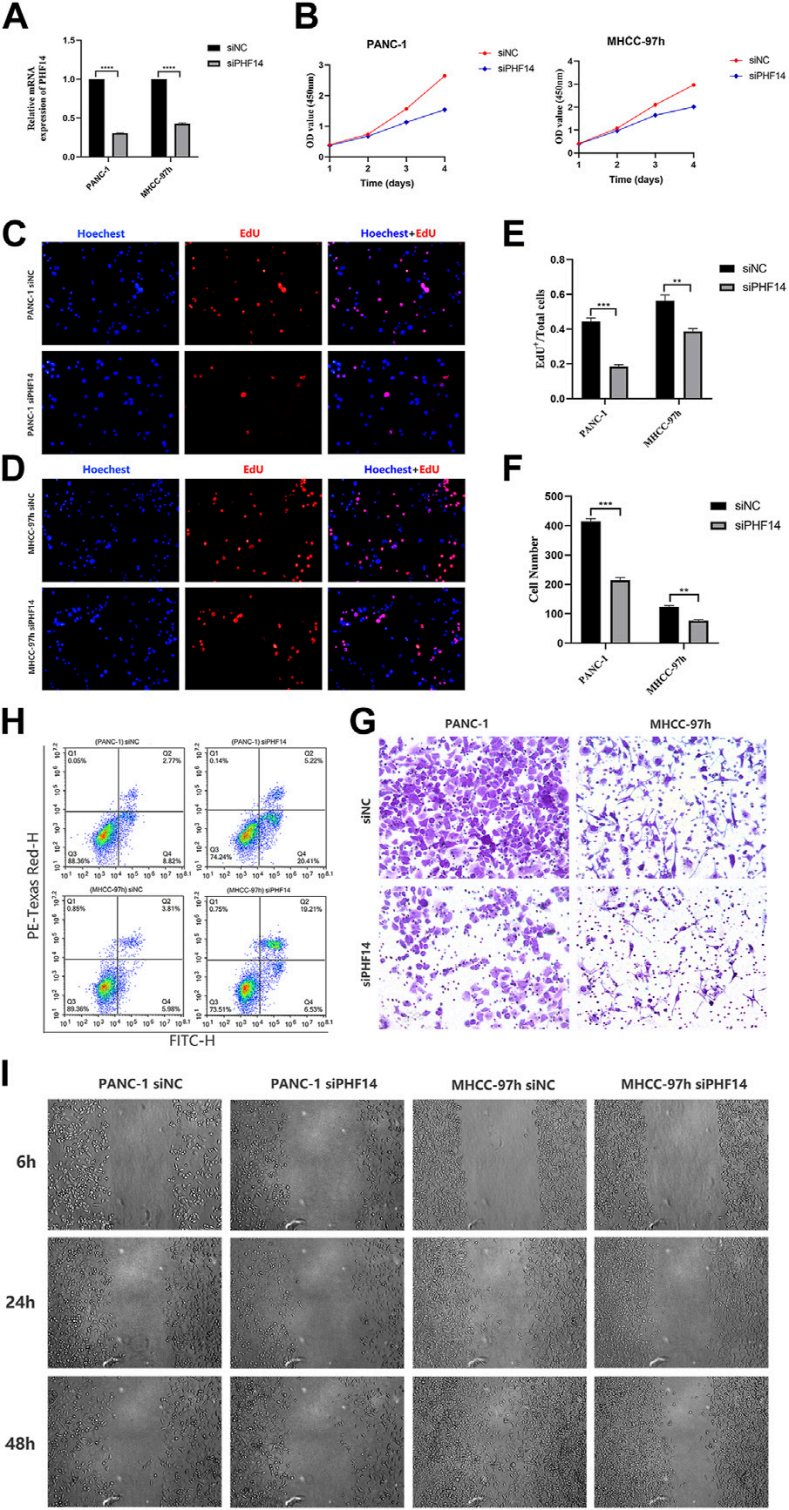
PHF14-knockdown inhibited the growth, migration and invasion of PANC-1 and MHCC-97h cells, and promoted cells apoptosis. **(A)** The knockdown efficacy of PHF14 examined in PANC-1 and MHCC-97h cells by qRT-PCR. **(B)** The effect of the siRNA targeting of PHF14 on cell proliferation was measured with the MTT assay at the indicated times following transfection. **(C, D)** Ten thousand PANC-1 and MHCC-97h cells per well were seeded in 96-well plates overnight. EdU and Hoechest co-staining was performed on the second day to assess cellular DNA replication activities (Scale bar = 200 μm). **(E)** Values are counted as EdU+ cells/total cells. **(F, G)** Cell suspensions (the cell density was adjusted to 1-10*10^5^/mL) of 200 μL PANC-1 and MHCC-97h were seeded into the upper chamber of a transwell insert (Scale bar = 100 μm). After 24 h of incubation, cells which had adhered to the lower membrane of the inserts were fixed, stained with 1% crystal violet and counted for analysis. **(H)** Flow cytometry was used to detect cell apoptosis in PHF14- and negative control-knockdown cells through annexin V-FITC/PI staining. **(I)** Wound closure scratch was used to assess the effect of PHF14 on the migratory capacity of PANC-1 and MHCC-97h cells. Each assay was performed in triplicate (**p < 0.01; ***p < 0.001; ****p < 0.0001).

To determine whether defective expression of PHF14 had a functional role in PANC-1 and MHCC-97h cells, lentivirus siRNA was used to knockout PHF14 expression in cells (Figure 9A). Two or three days after transfection, the PHF14-knockdown led to a downregulation of cell growth as determined *via* the MTT assay (Figure 9B). Subsequently, EdU assay showed that the growth of PANC-1 and MHCC-97h cells decreased after PHF14 gene silencing (Figures 9C–E).

The results of the transwell assay revealed that invasion potential of PANC-1-siPHF14 and MHCC-97h-siPHF14 cells were decreased relative to control cells (siNC) in transwell based experiment (Figures 9F, G). 48 h after transfection, the flow cytometry showed that the percentage of apoptotic cells was higher (25.63% versus 11.59%, and 25.74% versus 9.79%) than the control cells in PANC-1 and MHCC-97h short interfering PHF14 cells (Figure 9H). Wound closure scratch was used to assess the effect of PHF14 on the migratory capacity of PANC-1 and MHCC-97h cells. In contrast to control cells, cells with PHF14-knockdown had a significantly lower percentage of gap closures in the scratch assay (Figure 9I).

## Discussion

As an integral member of the newly discovered PHD family, PHF14 has been found to be responsible for multiple carcinogenesis and development, including colon cancer, biliary tract cancer, lung and bladder cancer (Ivanov et al., 2007; Akazawa et al., 2013; Pu et al., 2018). Huang’s findings revealed that PHF14 exerts a major effect in epigenetic modification and regulation by interacting with histones through its PHD1 and PHD3 structural domains. However, how PHF14 functions in the pathogenesis of different tumors through a number of common or similar molecular mechanisms is pending further investigation. Therefore, TCGA, CPTAC, and GEO databases were adopted to assess PHF14 expression, genetic alterations, survival prognosis or immune infiltration of 33 different cancer types. In this study, novel approaches were elucidated for the pathogenesis and clinical prognosis of PHF14 in various tumors.

In most TCGA tumors, PHF14 mRNA expression was significantly increased compared with adjacent normal tissues. Protein and target gene expressions of PHF14 were also correspondingly increased in these tumors, indicating the functional activity of PHF14 in these tumors. Wu et al. (Barrett et al., 2013; Zhang et al., 2019) conducted immunohistochemical analysis on 5 normal brain samples and 3 tissue microarrays of 105 glioma samples. The findings suggested an upregulation of PHF14 expression in glioma. Additionally, silencing PHF14 gene could effectively suppress the migration, invasion and proliferation of glioma cells, and promote cell apoptosis. Similarly, Zhao et al. (Ivanov et al., 2007; Akazawa et al., 2013; Pu et al., 2018) explored PHF14 expression level in gastric cancer tissues, and determined that PHF14 was highly expressed in gastric cancer. Moreover, the growth of tumor cells with PHF14 knockout was significantly inhibited.

In this study, we found that the prognostic data varied with different tumors and PHF14. Therefore, the relationship between PHF14 expression and survival in patients with different tumor types was further analyzed using the GEPIA2 tool. The findings revealed that the poor prognosis of OS was closely associated with high PHF14 expression in patients with ACC, COAD, LGG, SARC and UVM, whereas the opposite was true in CHOL. In recent years, several studies have revealed an association between PHF14 expression and decreased overall survival in STAD, BLCA, LUAD, GBM and LGG, while PHF14 overexpression suppressed the growth of tumor cells in CHOL and COAD (Ivanov et al., 2007; Akazawa et al., 2013; Pu et al., 2018; Wu et al., 2019; Zhao et al., 2020). However, little has been reported about the correlation between PHF14 expression and survival prognosis of other tumors.

For LUAD and CHOL, despite the similar results to previous studies, no correlation between PHF14 expression and survival prognosis in patients with STAD or BLCA was not observed in the TCGA project, and our findings on LUAD and COAD were also inconsistent with previous studies. One possible reason for such different results can be attributed to different data processing or updated survival information. In addition, using the Kaplan-Meier plotter for survival analysis, the highly expressed PHF14 was relevant to a poor DFS prognosis in ACC, COAD, KIRC, LGG, LIHC, LUAD, MESO cancer patients, while TGCT was the opposite. Simultaneously, the highly expressed PHF14 was significantly relevant to poor PFS prognosis in KIRC, LGG, LUAD, and PAAD cancers. It is further revealed that amplification or mutation is the most common type of alteration of PHF14 in most types of tumors, and the alteration of PHF14 is associated with poor prognosis of OS, DSS and PFS in cancer patients through the cBioPortal tool. Therefore, we speculate that the poor prognosis of the above tumors is most likely due to the expression or alteration of the PHF14 gene in them.

Although our study and some other previous studies have found that the relationship between PHF14 expression and survival prognosis varied when it comes to various tumors, we still believe that aberrant PHF14 gene expression has an adverse influence on the survival prognosis of the vast majority of tumors. To this end, an in-depth molecular experimental evidence is essential in confirming whether the highly expressed PHF14 is only the result of normal tissue resistance to tumor progression, and whether it has a major effect on the development of different tumors.

It is reported that, in the tumor micro-environment (TME), cancer-associated fibroblasts (CAFs) are heavily involved in the regulation of tumor-infiltrating immune cell functions, thus exerting a critical influence in coordinating tumor development (such as proliferation, invasion, migration and metastasis) (Ishii et al., 2016; Bu et al., 2019; Sahai et al., 2020). Therefore, multiple methods were used to study the relationship between cancer-related fibroblasts and PHF14 gene expression in various cancer types in TCGA, so as to better understand how PHF14 expression reacted tumor-infiltrating immune cells. Notably, it can be revealed from this study that PHF14 expression in most cancer types in TCGA exhibited positive relevance to the infiltration level of cancer-associated fibroblasts, especially in CESC, COAD, HNSC, KIRC, LUAD, PAAD, READ and SKCM, which was similar to survival analysis. PHF14 possibly exerts an effect on the survival status of patients by modifying the immune cell infiltration in the tumor microenvironment, such as cancer-associated fibroblasts, which requires further investigation.

In addition, as a membrane protein, immune checkpoint protein contribute to immune homeostasis mainly by mediating the activation of immune cells (Singh et al., 2020).

Therefore, how PFH14 expression interacted with immune checkpoint gene expression levels was also explored. Previous studies have revealed that the abnormal expression of immune checkpoint proteins is an essential mechanism of tumor immune escape (Wilky, 2019). Furthermore, He et al. (2015) also reported that immune checkpoint inhibitors (ICIs) can destroy cancer cells by enhancing and activating the immune system during the normal immune processes, which is similar to the results of our study. It was shown that PFH14 expression was correlated positively with tumor immune checkpoint gene expression levels, suggesting that PFH14 may affect tumor immunity through regulating immune checkpoint gene expression levels, thereby affecting tumor progression.

In this study, a series of enrichment analyses were conducted on the mixed information of PHF14-binding proteins and PHF14 expression-related genes in all tumors. The results suggested a potential role of “histone binding,” “nuclear chromatin,” “RNA splicing,” “histone modification” and “chromatin remodeling” in cancer etiology or pathogenesis. According to previous studies, PHF14, as a regulator and an essential member of the PHD finger family, acted as a key factor in the recognition and translation of histone modification marks (Wysocka et al., 2006; Lan et al., 2007; Soliman and Riabowol, 2007; Ballare et al., 2012; Huang et al., 2013; Zheng et al., 2021; Pan et al., 2022). Also, in our PHF14 pan-cancer analysis, the single genome enrichment analysis of sample subsets characterized by high and low expression of PHF14 showed that, PHF14 can be involved in a wide range of metabolic pathways, such as reactome gpcr ligand binding, reactome neuronal system, and naba core matrisome. However, there was a wide distribution of the highest enrichment fractions of these signalling pathways in both high and low expression regions of PHF14, suggesting that high or low expression of PHF14 was involved in regulating these signaling pathways in different tumors. From some previous studies, Wu et al. inhibited the Wnt signaling pathway by silencing the expression of PHF14 in glioblastoma multiforme, indicating that PHF14 was involved in glioma pathogenesis through the Wnt/β-catenin signaling pathway (Soliman and Riabowol, 2007; Pan et al., 2022). However, Zhao et al. investigated the growth of gastric cancer tissue by knocking down or not knocking down PHF14, and found that PHF14 contributed to the proliferation and migration of gastric cancer cells through the AKT and ERK1/2 pathways (Soliman and Riabowol, 2007; Pan et al., 2022). In addition, Miao et al. (2019) found that LINC00612 significantly affected the proliferation and invasion of tumor cells by sponging Mir-590 and PHF14 in bladder cancer tissues, indicating that PHF14 fuctioned as a key factor in different cancers through multiple signaling pathways or chromatin complex effects and other mechanisms. These findings may further shed light on the effect of PHF14 gene in the etiology or pathogenesis of different cancers.

In general, the first pan-cancer analysis of PHF14 conducted in this study clearly reveals that PHF14 can be expressed in most types of cancers and its expression is related to the clinical prognosis, genetic alterations, signaling, and immune cell infiltration in cancer patients, which contributes to a thorough insight into the tumorigenesis or potential mechanisms of PHF14.

## Conclusion

PHF14 expression level is highly relevant to carcinogenesis and prognosis of tumors. Yet, additional experiments are still necessary to further validate this conclusion and to explore the specific mechanisms in more detail.

## Data Availability

Publicly available datasets were analyzed in this study. This data can be found here: The UCSC XENA database (https://xenabrowser.net/datapages/), the timer database (https://cistrome.shinyapps.io/timer/), CCLE database (https://portals.broadinstitute.org/), the Human Protein Atlas (HPA) database (version: 20.1) (https://www.proteinatlas.org/), the UALCAN portal (http://ualcan.path.uab.edu/analysis-prot.html), the TCGA Pan-Cancer datasets (version: 2018–09–13) (https://xenabrowser.net/), the cBioPortal (version: 3.6.20) (https://www.cbioportal.org/), the Tumor Immune Estimation Resource version 2 (TIMER2) (http://timer.cistrome.org/), the STRING tool (https://string-db.org/).

## Glossary

PHF14: plant homeodomain finger protein 14
PHD: plant homeodomain
TCGA: the Cancer Genome Atlas
GEO: gene expression omnibus
BTC: biliarytract cancer
GTEx: genotype-tissue expression
CCLE: cancer cell line encyclopedia
HPA: human protein atlas
ACC: adrenocortical carcinoma
BLCA: bladder urothelial carcinoma
BRCA: breast invasive carcinoma
CESC: cervical squamous cell carcinoma
CHOL: cholangiocarcinoma
COAD: colon adenocarcinoma
DLBC: lymphoid neoplasm diffuse large B cell lymphoma
ESCA: esophageal carcinoma
GBM: glioblastoma
LGG: brain lower grade glioma
HNSC: head andneck squamous cell carcinoma
KICH: kidney chromophobe
KIRC: kidney renal clear cell carcinoma
KIRP: kidney renal papillary cell carcinoma
LAML: acute myeloid leukemia
LIHC: liver hepatocellular carcinoma
LUAD: lung adenocarcinoma
LUSC: lung squamous cell carcinoma
MESO: mesothelioma
OV: ovarian serous cystadenocarcinoma
PAAD: pancreatic adenocarcinoma
PCPG: pheochromocytoma and paraganglioma
PRAD: prostate adenocarcinoma
READ: rectum adenocarcinoma
SARC: sarcoma
SKCM: skin cutaneous melanoma
STAD: stomach adenocarcinoma
TGCT: testicular germ cell tumors
THCA: thyroid carcinoma
THYM: thymoma
UCEC: uterine corpus endometrial carcinoma
UCS: uterine carcinosarcoma
UVM: uveamelanoma
CPTAC: clinical proteomic tumor analysis consortium
GEPIA2: GeneExpression profile interactive analysis version 2
OS: overall survival
DFS: disease-free survival
K-M: Kaplan-Meier
PFS: progression-free survival
CNA: copy numberalteration
TIMER2: tumor immune estimation resource version 2
CAFs: cancer associated fibroblasts
GDC: genomic data commons
GO: gene ontology
GSEA: gene set enrichment analysis
KEGG: Kyoto encyclopedia of genes and genomes
