# Supplementary figures and images for: A comprehensive pan-cancer analysis unveiling the oncogenic effect of plant homeodomain finger protein 14 (PHF14) in human tumors
Source: Front Genet. 2023 Mar 10;14:1073138. doi: 10.3389/fgene.2023.1073138 (PMC10061232; doi:10.3389/fgene.2023.1073138)

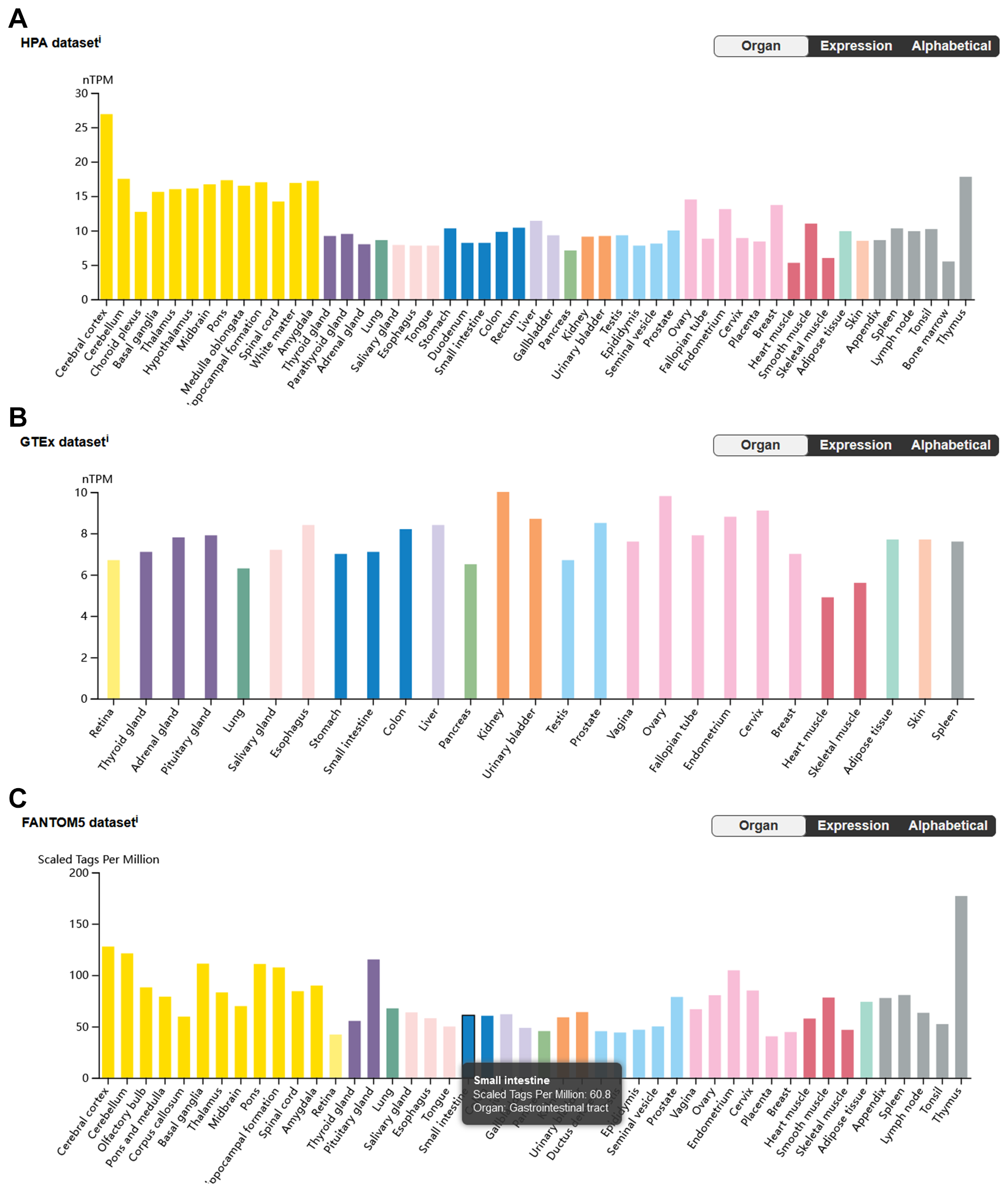

Supplement: Supplementary file 1 [file Image1.tif]
